# Medical and midwifery students’ views on the use of conscientious objection in abortion care, following legal reform in Chile: a cross-sectional study

**DOI:** 10.1186/s12910-020-00484-4

**Published:** 2020-05-24

**Authors:** M. Antonia Biggs, Lidia Casas, Alejandra Ramm, C. Finley Baba, Sara P. Correa

**Affiliations:** 1 Advancing New Standards in Reproductive Health (ANSIRH), Bixby Center for Global Reproductive Health, Department of Obstetrics, Gynecology and Reproductive Sciences, University of California, San Francisco (UCSF), 1330 Broadway, Suite 1100, Oakland, CA 94612 USA; 2 Centro de Derechos Humanos, Facultad de Derecho, Universidad Diego Portales, Santiago, Chile; 3 Instituto de Investigación en Ciencias Sociales, Universidad Diego Portales, Santiago, Chile; 4 Escuela de Sociología, Universidad de Valparaíso, Valparaíso, Chile

## Abstract

**Background:**

In August 2017, Chile lifted its complete ban on abortion by permitting abortion in three limited circumstances: 1) to save a woman’s life, 2) lethal fetal anomaly, and 3) rape. The new law allows regulated use of conscientious objection (CO) in abortion care, including allowing institutions to register as objectors. This study assesses medical and midwifery students’ support for CO, following legal reform.

**Methods:**

From October 2017 to May 2018, we surveyed medical and midwifery students from seven universities located in Santiago, Chile. Universities included 4 secular (2 public and 2 private) and 3 private religiously-affiliated universities; all offering medical degrees with a specialization in obstetrics and gynecology (ob-gyn) and five offering midwifery degrees. We used generalized estimating equations (GEE) to identify characteristics associated with student support for CO, intentions to use CO to refuse to care for someone seeking abortion, and support for CO at the institutional level.

**Results:**

333 of the 413 eligible students who opened the survey, completed the questions on conscientious objection; 26% were seeking medical degrees with an ob-gyn specialty, 25% were seeking midwifery degrees, and 49% were seeking medical degrees and had not yet decided their specialty. While nearly all endorse requirements for conscientious objecting clinicians to inform (92%) and refer (91%) abortion-seeking patients, a minority (18%) would personally use conscientious objection to avoid caring for a patient seeking abortion (12% secular and 39% religious university students). About half of religious-university students (52%) and one-fifth of secular-university (20%) students support objections at the institutional level.

**Conclusions:**

Most students support the regulated use of CO which preserves patients’ access to abortion care. Religious-university student views on the use of conscientious objection in abortion care are discordant with those of their institutions which currently support institutional-level objections.

## Background

Conscientious objection emerged as a means to allow people to refuse to participate in military service due to personal beliefs. It has expanded to also allow health care professionals to refuse to provide reproductive health care, and in particular abortion care [1–3]. However, the assumption that conscience-based refusals need to be accommodated in abortion care has recently been challenged [4–6]. Some contend that it is unethical to deny people access to health care on the basis of non-verifiable personal, non-evidence based beliefs and that when health care professionals choose their profession they are agreeing to the professional obligation to serve their patients, unlike mandatory military service [7].

It has been argued that conscientious objection may be used for reasons other than conscience, such as avoiding the stigma of providing abortion care, avoiding participating in care where one has limited training, and to reduce one’s workload [3, 8]. In Latin America, there is the idea of a “double discourse” where in public, individuals such as health professionals, uphold the prevailing, highly restrictive cultural norms, whereas in private, their views are much less conservative [9]. This “double discourse” might compel health professionals in Latin America to publically declare themselves as objectors, even if not in accordance with their private views. Studies have shown that widespread use of conscientious objection can limit access to abortion care, particularly for those living in rural areas [10, 11]. In an effort to preserve people’s access to abortion care, international professional organizations have provided guidance aimed at ensuring that the rights of the health professional are balanced with those of the patient seeking abortion [5, 12–14]. It is also widely accepted that health professionals have the professional responsibility to treat patients seeking post-abortion care, irrespective of their personal views about abortion [15].

In 2017, Chile lifted its complete ban on abortions, permitting abortions: 1) to save a woman’s life, 2) for lethal fetal anomalies, and 3) due to rape [16]. The current law requires that all people seeking an abortion be given information about social and financial support services, referrals to a willing provider, and offered psychological support services. The health professional’s role in the abortion procedure is not yet clearly defined. Only physicians are legally allowed to provide an abortion, yet midwives often care for the patient. In the case of medication abortion, physicians write the prescription and midwives might inform and give the patient the prescription. Abortions can only take place in hospitals or clinics with high risk obstetric units, not private practices, even though abortion is not a high risk procedure. All personnel present during the abortion procedure, including physicians, midwives, anesthetists, and nurses can claim conscientious objection refusals. Objecting providers must register as objectors, offer patients information and referrals to a non-objecting provider, and if a non-objecting provider is not available, provide abortion care in the case of a life-threatening emergency [17]. When registering as an objector, the provider must first notify the hospital or clinic director in writing before they can object to an abortion procedure and they must indicate for which of the three legal grounds (i.e. abortions to save a woman’s life, lethal fetal anomaly, or rape) they object. Registration must occur before a patient requests the abortion. The hospital or clinic director must honor the objecting provider’s status. The goal of the registry is to facilitate referral practices by documenting the number of providers willing to do a procedure in each facility. The law does not allow conscience-based refusal claims for pre-abortion (diagnosis) or post-abortion care. A recent survey indicates that 47% of ob-gyn physicians working in the 69 public hospitals designated to provide abortions claim conscientious objection to care for women seeking abortions due to rape, 27% claim refusals for abortions due to fetal anomalies, and 20% claim refusals to save a woman’s life [18]. Midwives represent 21% of people registered as objectors to participating in abortions due to rape, 18% of objectors for abortions due to fetal anomaly, and 12% of objectors for abortions to save the woman's life [19].

Like other countries in Latin America, Chile allows private, but not public institutions to claim objector status [17]. Chile’s Catholic University “Pontificia Universidad Católica”, which houses the country and region’s top medical school and includes a wide network of hospitals and health centers, became the first institution to claim objector status at the institutional level. While claiming themselves objectors at the institutional level does not preclude them from training health professionals in abortion care, they have not indicated that legal reform would result in changes to their existing curriculum. Furthermore, in interviews with medical and midwifery school faculty, most of those working at religiously-affiliated universities described strong support for the use of conscientious objection at the institutional and individual level and did not believe their curriculum needed to change in response to legal reform [20]. However, a recent survey of medical and midwifery university students from both religiously-affiliated and secular universities found that most support recent abortion decriminalization, believe their university should train medical and midwifery students to provide abortion care, and are interested in becoming trained to provide abortion services [21, 22]. The current study, builds on these findings by assessing these same students’ support for the use of conscientious objection in abortion care.

## Methods

### Recruitment procedures

We reviewed the Chilean Ministry of Education website and identified seven universities that offer midwifery or medical degrees with a specialization in obstetrics and gynecology (ob-gyn), located in Santiago, to serve as recruitment sites. We included a mix of public, private, secular and religiously-affiliated universities (all of which were Catholic), with a relatively high volume of students seeking degrees in medicine with an ob-gyn specialty or midwifery. All seven universities have a medical school and five a midwifery department. We estimate that the seven participating universities serve over 7000 students seeking medical or midwifery degrees, representing 72% of medical and 38% of midwifery students in the metropolitan region of Santiago and 36% of medical and 16% of midwifery students in the country [23].

Research staff emailed department administrators and student council leaders requesting them to distribute a survey link to their medical and midwifery students. The survey items related to conscientious objection were drawn from an instrument designed to measure conscientious objection to abortion provision among practicing clinicians [24] and modified to be applicable to university students. Six departments at four universities shared the link with students directly, through student listservs or department Facebook pages. At the two universities (four departments) that did not respond to emails, research staff distributed small paper flyers that included the survey link and a QR code to medical and midwifery students. Before completing the survey, eligible and interested students first reviewed the online consent form and gave written consent. At the end of the survey, we gave participants the option to enter into a drawing for a gift card worth $40 USD/24,000 Chilean pesos and randomly selected 25 winners. We collected student surveys from October 2017 to May 2018, shortly after legal reform in August 2017. This study received human subjects’ approval from the Committee of Ethics of the Institute of Social Science Research at the University of Diego Portales (UDP) located in Santiago, Chile.

We powered our sample to detect mean differences in abortion attitudes by university type (secular vs religious university) and degree type (medical vs midwifery). We estimated that a sample of 300, with a minimum group size of 90, could detect a mean difference of 0.45, on a 4-point scale, and as reported in a published abortion stigma subscale, with a standard deviation of 1.07, and a two-sided alpha of 5 and 80% power [25].

### Analyses

We examined students’ views about requirements for clinicians who invoke conscientious objection in abortion care and whether they personally support or would use conscientious objection (CO) to avoid caring for a patient. While most questions were specific to the use of CO in abortion care, some were about the use of CO generally. Specifically, we examined responses to eight Likert-scaled items (ranging from strongly agree to strongly disagree which included whether objecting providers should be: 1) required to counsel patients with unwanted pregnancies on all treatment options, including abortion, 2) required to refer patients eligible for a lawful abortion to a willing clinician, or 3) allowed to refuse to provide post-abortion care; whether they support 4) mandatory public registration of objecting providers, 5) allowing universities and other institutions to register as conscientious objectors, and whether they would use conscientious objection to avoid caring for a woman 6) who wanted an abortion, no matter what her reasons, 7) who wanted a lawful abortion, or 8) with post-abortion complications.

For multivariable logistic regression analyses, these eight items were dichotomized (strongly agree or agree vs neither agree nor disagree, disagree, and strongly disagree). We used logistic general estimating equation (GEE) models, and selected model covariates a priori. These included respondent characteristics that are known to be associated with abortion attitudes, based on the existing literature [26, 27]. Specifically, we included university type (secular or religiously-affiliated), gender, age group, type of degree sought (medical-undecided specialty, medicine-obstetrics and gynecology specialty, and midwifery), year in medical/midwifery school, region where student completed high school (Santiago metropolitan region vs other), religion (Catholic or other religion vs no religious affiliation), frequency of attendance to religions services, and political affiliation, which included none/Center (moderate), Right/Center right (conservative), and Left/Center left (progressive). Analyses accounted for clustering by university. We conducted all analyses in STATA 14 and report significance at *P* ≤ .05. We excluded missing responses on any outcomes from all analyses.

## Results

### Respondent characteristics

Of the 459 students who opened the survey link, we removed 46 responses due to ineligibility and 80 due to incomplete responses, leaving a final sample of 333, a completion rate of 81% (333/413 eligible participants). There were no statistically significant differences by gender, religion, age, year in school, type of school, or field of study between the final sample (*n* = 333) and those with incomplete surveys. However, participants with a center or no political affiliation were significantly (*p* < .05) less likely to complete the survey than those who identified as left/center left or right/center right. The majority of students were attending a secular (77%) university and seeking a medical degree (75%, Table 1). Approximately one quarter (26%) were seeking a medical degree and planning to specialize in ob-gyn and one-quarter (25%) were seeking a midwifery degree, most of whom were female (94%).

**Table 1:** Participant characteristics

	Number	Percent
**Total**	**333**	**100**
Female	209	63
Age group		
17–19	80	24
20–24	197	59
25–37	56	17
Type of degree being pursued		
Midwifery	83	25
Medicine-Planning to specialize in Obstetrics/Gynecology	86	26
Medicine-Specialty undecided/unknown	164	49
Year in medical or midwifery school		
1st-2nd	146	44
3rd-4th	108	32
5th–7th/Just graduated	79	24
Born in Chile	326	98
Lived one year or more outside of Chile	19	6
Region where completed high school		
Santiago metropolitan region	259	78
Northern Chile	24	7
Southern Chile	47	14
Other country	3	< 1
Single/not married	327	98
Political affiliation		
Right/center right	81	24
Center	27	8
Left/center left	158	48
None	67	20
Frequency of religious service attendance		
Once a week/2–3 times a month	42	13
Once a month/2–3 times a year	50	15
Hardly ever/never	237	72
Religion		
Catholic	121	36
Evangelical/Protestant	15	5
Other	15	5
None/Atheist/Agnostic	182	55
University type		
Private-Secular	138	41
Private-Religious	71	21
Public-Secular	124	37

### Views about conscientious objection

Nearly all students agreed/strongly agreed that clinicians who conscientiously object should counsel women with unwanted pregnancies on all their treatment options, including abortion (92%) (Table 2); that they should refer women eligible for a lawful abortion to a clinician willing to provide it; one-fifth agreed/strongly agreed that clinicians should be allowed to refuse to provide post-abortion care (18%), yet only 6% said they would invoke conscientious objection to avoid caring for a woman with post-abortion complications and 18% would invoke conscientious objection to avoid caring for a woman seeking abortion. Nearly two-thirds (67%) agreed that mandatory public registration of conscientious objectors should be implemented in Chile, yet only a little over one-quarter (27%) agreed that universities and other institutions should be able to do so. Students from religiously-affiliated universities were significantly (*p* < .05) more likely to agree that they would use conscientious objection in certain circumstances than students attending secular universities.

**Table 2:** Proportion of students who agree/strongly agree with statements about conscientious objection (CO), by university type (N = 333)

	Total %	University type, %	
		Religious	Secular
Clinicians who conscientiously object should:			
Counsel patients with unwanted pregnancies on all their treatment options, including abortion	92	75	97*
Refer patients eligible for a lawful abortion to a willing clinician	91	76	95*
Be allowed to refuse to provide post-abortion care	18	25	17
I would use CO to avoid caring for a woman:			
who wanted an abortion, no matter what her reasons	18	38	12*
who wanted a lawful abortion	18	39	12*
with post-abortion complications	6	10	5
The following CO regulations should be implemented in Chile:			
Universities and other institutions should be able to register as COs	27	52	20*
Mandatory public registration of COs	67	64	68
The following health professionals should be able to be COs:			
Physicians	83	97	79*
Midwives	79	96	75*
Nurses	62	80	56*
Pharmacists	32	55	26*
Administrators	23	38	19*
Nobody should be able to conscientiously object	16	3	20*

* Differences between students attending religious and secular universities are statistically significant (*p* < .05)

In multivariable analyses, factors associated with endorsing the view that a clinician who conscientiously objects should counsel women with unwanted pregnancies on all of their treatment options include type of university and degree, political affiliation, and religious attendance (Table 3). Secular-university students had higher odds of endorsing this view (aOR: 4.57; 95% confidence interval [CI]: 1.44, 14.48). Students pursuing a midwifery degree (aOR: 0.16; 95% CI: 0.04, 0.74), whose political affiliation was right/center right (aOR: 0.24; 95% CI: 0.07, 0.86) and who frequently attended religious services (aOR: 0.13; 95% CI: 0.03, 0.54), had significantly lower odds of endorsing this view when compared to their counterparts. Secular-university students (aOR: 3.72; 95% CI: 1.36, 10.12), women (aOR: 3.22; 95% CI: 1.14 9.09) and students who attended high school in Santiago (aOR: 2.77; 95% CI: 1.03, 7.47) had higher odds of agreeing that clinicians who claim conscientious objection should refer women to a willing provider. Students who frequently attended religious services (aOR: 0.27; 95% CI: 0.08, 0.96) had significantly lower odds of endorsing this view.

**Table 3:** Multivariable logistic regression analyses assessing factors associated with agreeing (agree/strongly agree) with statements about conscientious objection (CO)

Respondent characteristics	Clinicians who invoke CO should:						Regulations that should be implemented:			
	...counsel patients on all treatment options		...refer patients to a willing clinician		…be allowed to refuse to provide post-abortion care		Universities & other institutions should be able to register as COs		Mandatory public registration of COs	
	%	aOR[95% CI]	%	aOR[95% CI]	%	aOR[95% CI]	%	aOR[95% CI]	%	aOR[95% CI]
University type										
Secular	97	4.57*[1.44,14.48]	95	3. 72*[1.36,10.12]	17	0.87 [0.40,1.89]	20	0.37*[0.17,0.80]	68	0.72 [0.36,1.45]
Religious (Ref.)	75		76		25		52		64	
Gender										
Female	92	2.15 [0.63,7.29]	94	3.22*[1.14,9.09]	22	1.81 [0.90,3.66]	27	0.85 [0.42,1.70]	68	1.22 [0.71,2.11]
Male/Other (Ref.)	92		86		12		26		64	
Age group										
17–19	93	1.07 [0.22,5.12]	89	0.36 [0.08,1.53]	21	0.67 [0.30,1.52]	26	1.15 [0.45,2.91]	71	0.84 [0.39,1.80]
20–24 (Ref)	92		92		19		22		67	
25–37	91	1.90 [0.32,11.23]	91	1.01 [0.18,5.62]	13	0.95 [0.32,2.84]	42	4.77*[1.75,13.06]	62	0.95 [0.43,2.10]
Degree being pursued										
Medicine-Specialty undecided/unknown (Ref.)	92		88		16		23		69	
Midwifery	86	0.16*[0.04,0.74]	93	1.23 [0.31,4.97]	27	1.10 [0.51,2.38]	33	2.39*[1.00,5.71]	71	0.91 [0.45,1.84]
Medicine-Planning to specialize in OB-GYN	97	2.66 [0.54,13.04]	94	1.86 [0.54,6.35]	15	0.79 [0.37,1.69]	27	1.78 [0.82,3.90]	59	0.56[0.31,1.02]
Where completed high school										
Santiago metropolitan region	92	1.59 [0.49, 5.20]	92	2.77*[1.03,7.47]	18	0.92 [0.46,1.87]	24	0.39*[0.19,0.80]	67	0.98 [0.55,1.78]
Other location (Ref.)	92		86		19		36		65	
Political affiliation										
Center/None (Ref.)	95		89		19		32		69	
Right/Center right	77	0.24*[0.07,0.8]	79	0.76 [0.26,2.21]	24	1.30 [0.56,3.00]	53	1.39 [0.63,3.09]	51	0.47*[0.23,0.95]
Left/Center left	98	0.93 [0.17,5.10]	98	3.74 [0.90,15.56]	15	0.99 [0.46,2.12]	10	0.37*[0.17,0.80]	74	1.31 [0.71,2.44]
Religion										
Catholic or other religion (Ref.)	98		96		23		42		61	
None	85	1.52 [0.31,7.62]	85	1.80 [0.47,6.95]	14	0.74 [0.34,1.62]	14	0.57 [0.26,1.26]	72	1.38 [0.71,2.68]
Frequency of religious attendance										
Hardly ever/never (Ref.)	97		95		16		18		70	
Once a month/2–3 times a year	88	0.79 [0.16,3.82]	88	1.11 [0.26,4.76]	24	1.21 [0.47,3.09]	33	0.84 [0.33,2.18]	61	0.94 [0.42,2.10]
Once a week/2–3 times a month	67	0.13*[0.03,0.54]	69	0.27*[0.08,0.94]	24	1.22 [0.47,3.08]	70	5.25*[2.03,13.56]	60	1.09 [0.47,2.54]
Year in school										
1st-2nd (Ref.)	92		92		24		26		71	
3rd-4th	94	0.75 [0.15,3.78]	89	0.43 [0.10,1.90]	17	0. [0.24,1.16]	25	1.06 [0.43,2.57]	65	0.70 [0.35,1.42]
5th–7th/just graduated	90	0.22 [0.03,1.70]	92	0.84 [0.11,6.31]	11	0.37 [0.13,1.14]	28	0.62 [0.19,2.04]	63	0.73 [0.31,1.71]

*aOR* Adjusted odds ratios, *CI* Confidence Intervals; * *p* < .05; Ref = Referent group

Factors associated with higher odds of agreeing that universities and other institutions should be able to register as conscientious objectors included being ages 25–37, pursuing a midwifery degree, and frequent religious attendance. Attending a secular university, completing high school in Santiago’s metropolitan region, and identifying as center left/left political affiliation were associated with lower odds of agreeing with this view. Political affiliation was the only factor associated with agreeing that there should be mandatory registration of conscientious objectors; students identifying as right/center right had significantly lower odds of agreeing with this view (aOR: 0.47; 95% CI: 0.23, 0.95).

When asked whether they agree with the statements about using conscientious objection to avoid caring for women who wanted an abortion “no matter what her reasons” or for a woman who “wanted a lawful abortion”, students whose political affiliation was right/center right (43 and 46%, respectively) and who frequently attended religious services (54 and 56%, respectively) had higher odds of agreeing with these statements (Table 4). When compared to medical students with an unknown specialty (21%), those specializing in ob-gyn (11%) had lower odds of agreeing with the statement that they would use conscientious objection to avoid caring for a woman who wanted a lawful abortion (aOR: 0.35; 95% CI: 0.13, 0.93). Midwifery students (14%) had nearly seven times higher odds than medical students with an unknown specialty (4%) to agree with the statement that they would use conscientious objection to avoid caring for a woman with post-abortion complications (aOR: 6.81; 95% CI: 1.53, 30.36).

**Table 4:** Multivariable logistic regression analyses assessing factors associated with personal support for invoking conscientious objection (CO)

Respondent characteristics	I would invoke CO to avoid caring for a woman:					
	who wanted an abortion, no matter what her reasons		who wanted a lawful abortion		with post-abortion complications	
	%	aOR[95% CI]	%	aOR[95% CI]	%	aOR[95% CI]
University type						
Secular	12	0.43*[0.19,0.96]	12	0.67 [0.30,1.52]	5	1.63 [0.44,6.08]
Religious (Ref.)	38		39		10	
Gender						
Female	20	1.66 [0.75,3.67]	18	1.02 [0.46,2.24]	8	1.04 [0.28,3.83]
Male/Other (Ref.)	14		18		4	
Age group						
17–19	15	0.97 [0.33,2.87]	16	0.82 [0.27,2.29]	5	0.46 [0.11,1.90]
20–24 (Ref)	17		17		6	
25–37	24	1.73 [0.56,5.31]	24	2.97 [0.91,9.74]	9	3.73 [0.69,20.17]
Degree being pursued						
Medicine-Specialty undecided/unknown (Ref.)	18		21		4	
Midwifery	22	1.22 [0.48,3.08]	20	0.73 [0.29,1.50]	14	6.81*[1.53,30.36]
Medicine-planning to specialize in OB-GYN	12	0.57 [0.22,1.46]	11	0.35*[0.13,0.93]	4	1.01 [0.22,4.68]
Where completed high school						
Santiago metropolitan region	16	0.53 [0.24,1.17]	18	1.00 [0.43,2.31]	7	1.59 [0.40,6.26]
Other location (Ref.)	23		19		4	
Political affiliation						
Center/None (Ref.)	15		16		6	
Right/Center right	43	3.09*[1.30,7.32]	46	4.04*[1.,9.60]	13	2.71 [0.77,9.60]
Center left/left	6	0.62 [0.24,1.62]	6	0.60 [0.23,1.59]	3	0.90 [0.22,3.63]
Religion						
Catholic or other religion (Ref.)	8		7		3	
None	29	0.59 [0.24,1.43]	32	0.43 [0.17,1.09]	10	0.65 [0.17,2.44]
Frequency of religious attendance						
Hardly ever/never (Ref.)	11		10		5	
Once a month/2–3 times a year	18	0.58 [0.19,1.75]	27	1.48 [0.53,4.13]	6	0.93 [0.18,4.81]
Once a week/2–3 times a month	54	3.81*[1.46,9.93]	56	4.60*[1.76,12.05]	18	2.78 [0.73,10.58]
Year in school						
1st-2nd (Ref.)	16		17		8	
3rd-4th	19	1.34 [0.49,3.68]	20	0.91 [0.34,2.44]	5	0.46 [0.11,1.83]
5th–7th/just graduated	19	0.96 [0.25,3.69]	17	0.26 [0.06,1.11.]	6	0.55 [0.08,3.75]

*aOR* Adjusted odds ratios, *CI* Confidence Intervals; *p < .05; Ref. = Referent group

## Discussion

We found moderate student support for the existence of conscientious objection in abortion care. A minority of students claimed they would invoke conscientious objection in practice (18%) and nearly all (92%) believed that conscientious objectors should be obligated to inform patients of all their treatment options and refer them to a willing clinician, a somewhat higher proportion than that reported among U.S. physicians (86% inform and 71% refer) [28]. There were also salient and expected differences in views by university type, with students from religiously-affiliated universities being more supportive of the use of conscientious objection and of reporting that they themselves would refuse to provide a woman abortion care. Consistent with other studies of students and physicians in other countries [26, 28, 29], agreement with the statement that they would invoke conscientious objection to refuse caring for a patient seeking abortion care was particularly high among students from religious universities, who identified with a right or far right political affiliation and among those who frequently attended religious services. Over one-third (38%) of students at religious universities claimed they would refuse to provide abortion care to a patient seeking it, which is likely to negatively impact patients’ access to abortion care. However, unlike what has been found in other studies [3], students reported that they were similarly likely to invoke conscience-based refusals whether for abortions permissible by law or for abortions due to other reasons, suggesting that their views about conscientious objection in abortion care are centered on the moral status of the fetus.

The vast majority of students across university type, support regulating the use of conscientious objection so that it does not compromise patients’ access to abortion care. Nearly all students from secular universities and three-quarters of religious-university students agree that an objecting provider should be required to provide patients information and referrals. These findings suggest that many students, in particular secular university students, support the “conventional compromise” proposed by Brock [30] whereby a clinician is permitted to conscientiously object to providing a medical intervention, but that they should remain obligated to refer patients to a willing provider, or otherwise leave the profession. We found that while some students were not willing to provide abortion-related care, the vast majority believe clinicians should facilitate people’s access to abortion, similar to what is required by law. However, as many as one in four students from religiously-affiliated universities support allowing objectors to refuse to offer information and referrals to a willing clinician, and to refuse to provide post-abortion care. These views are in conflict with the current law, as well as international ethical guidelines for ob-gyn physicians to promote health, well-being, and patient autonomy [13, 14]. Students may need clarification of their legal obligation to ensure patient access to health care services, including providing post-abortion care, irrespective of their personal views on abortion. Given that nearly half of ob-gyn providers in public hospitals currently refuse to provide care to people seeking abortion due to rape [31], broad support for conscientious objection among providers may pose a significant barrier to legal abortion care. We should continue to monitor whether the use of conscientious objection in Chile is placing undue pressure on the providers willing to provide abortion care or limit people’s access to abortion, as has been found in other countries [32–34].

Soon after legal reform, two highly prestigious religious universities in Chile registered as objectors at the institutional level, affirming that they will not provide an abortion to anyone within their institutions or affiliated private health centers and hospitals. Yet, in this study, only half of religious-university and one-fifth of secular-university students support institutional-level objections, and most students, even those from religious universities, believe their university should train their medical and midwifery students on abortion care [21]. Our findings suggest a mismatch between religious-university student views and those of their institutions which staunchly support institutional-level conscientious objection. Institutional-level objections may not only impact the availability of abortion services within the large number of hospitals and health centers affiliated with these religious universities, but also the content and availability of abortion training within these institutions. Students choose their programs mostly because of their prestige and location, not because of the institutions’ religious views practice [35]. The Catholic universities in Chile are among the most prestigious in the country and region, making them an esteemed destination of study, irrespective of students’ personal or religious views. The discordance between student views and administrative policies suggests that these universities may need to ensure that they are meeting the majority of their students’ desires to become trained to provide abortion care, and to consider offering students alternative training options at facilities that provide abortion care. Training in medical ethics, particularly in the topic of conscientious objection can provide students’ the opportunity to reflect on the relationship between their own moral values, professional duties and society.

This study had some limitations. Our response rate was low, although typical of what you would expect for an online survey of this type [36], our findings likely underrepresent people from religious universities and thus likely underrepresents views that are supportive of conscientious objection in abortion care [23]. According to the Chilean National Council of Education, approximately 35% of the medical and midwifery student population within our seven university recruitment sites are at religiously-affiliated universities, whereas less than one quarter (23%) of our responding sample came from religiously-affiliated universities [23]. Mitigating some concerns of bias, are the lack of statistically significant differences between participant characteristics and rates of survey completion. Nonetheless, the significant associations between variables should not be affected by non-response bias. Furthermore, because the students surveyed were not yet trained to provide clinical care, we only asked them whether they would refuse to “care for” a woman and not whether they would refuse “to provide” an abortion. The true proportion of students who might claim conscience-based refusals in abortion care in practice, is likely to be much higher. The strength of our research rests in that it captures students’ perspectives from both secular and religiously-affiliated universities, representing a wide spectrum of Chilean universities that offer degrees in medicine and midwifery. Another important strength lies in that we were able to document views about conscientious objection soon after legal reform.

## Conclusions

We find that most students support the regulated use of conscientious objection which preserves patients’ access to abortion care. However, about half of religious-university students do not support conscientious objection at the institutional level, suggesting discordance between student views and those of their institutions which currently support institutional-level objections. Medical and midwifery schools are key to building a workforce capable of providing people with timely, high quality, and nonjudgmental abortion services. Current training programs should ensure that these future health professionals have the knowledge and skills to navigate their own personal views about abortion while also addressing patients’ health care needs. Medical and midwifery schools are well positioned to ensure that all people have access to abortion services, while respecting the conscientious convictions of their students. Programs should focus on the ethical implications of denying people abortion care, in particular pre-abortion and post-abortion services. Future studies will need to examine whether students’ views on conscientious objection persist in practice.

## Supplementary information

**Additional file 1.**

## Data Availability

Data will be made available upon reasonable request.
