# Supplementary material for: Medical and midwifery students’ views on the use of conscientious objection in abortion care, following legal reform in Chile: a cross-sectional study
Source: BMC Med Ethics. 2020 May 24;21:42. doi: 10.1186/s12910-020-00484-4 (PMC7245938; doi:10.1186/s12910-020-00484-4)
Supplement: Supplementary file 1 — Additional file 1. [file 12910_2020_484_MOESM1_ESM.docx]

| ¡Muchas gracias por participar en nuestro estudio! Recuerde que sus respuestas a este cuestionario no serán vinculadas a ningún dato que pudiera identificarle. Puede saltar cualquier pregunta que no quiera contestar o que lo haga sentir incómodo/a.  Como agradecimiento al terminar de contestar puede participar de un sorteo de 25 tarjetas de regalo por 25.000 pesos.  No hay respuestas “correctas” o “incorrectas” - todo lo que diga es valioso e importante para nuestra investigación.  ¡Haga clic en la flecha para comenzar! | | |
| --- | --- | --- |
| SECCIÓN 1: DEMOGRÁFICAS | | |
| 1. **¿Cuántos años tiene? ___** | | |
| 1. **¿Cuál es su género?**    1. Mujer    2. Hombre    3. Otro: ______ | | |
| 1. **¿Dónde nació?**    1. En Chile    2. Otro país: ______ | | |
| 1. **¿Ha vivido más de un año fuera de Chile?**    1. No    2. Sí ¿Dónde?: ____________ | | |
| 1. **¿Cuál era la dependencia administrativa del establecimiento educacional en el que realizó su educación escolar?**    1. Municipal    2. Particular Subvencionado    3. Corporación de Administración Delegada    4. Particular Pagado | | |
| 1. **¿Qué carrera estudia?**    1. Obstetricia    2. Medicina    3. Otra: __________ | | |
| 1. **[Omitted for blinding]** | | |
| 1. **[*filtro: solo estudiantes de medicina*] ¿Piensa seguir la especialidad de Obstetricia y Ginecología?**    1. No    2. Sí    3. Quizás | | |
| 1. **¿Qué año de la carrera está cursando?**    1. Primer año    2. Segundo año    3. Tercer año    4. Cuarto año    5. Quinto año    6. Sexto año    7. Séptimo año    8. Otro: ___ | | |
| 1. **¿En qué región o país completó su enseñanza media?** | | |
| - 1. I   2. II   3. III   4. IV   5. V   6. VI | - 1. VII   2. VIII   3. IX   4. X   5. XI | - 1. XII   2. Región Metropolitana   3. XIV   4. XV   5. Otro país: __________ |
| 1. **¿Cuál es su estado civil?**    1. Soltero/a    2. Conviviente Civil    3. Casado/a    4. Separado/a (casado/a legalmente, pero separado/a, no vive con su esposo/a legal)    5. Divorciado/a    6. Viudo/a | | |
| 1. **En términos de afinidad política, ¿Se siente más cercano(a) a la…?**    1. Derecha    2. Centro-derecha    3. Centro    4. Centro- izquierda    5. Izquierda    6. Ninguna | | |
| 1. **¿Podría decirme cuál es la religión o iglesia a la que Ud. pertenece o se siente más cercano?**   [Seleccione todas las respuestas que correspondan]   - 1. Católica   2. Evangélica   3. Protestante   4. Judía   5. Otra: ­­­____________   6. Ninguna   7. Atea/o   8. Agnóstico/a | | |
| 1. **¿Con qué frecuencia asiste a la iglesia o practica su culto?**    1. Una o más veces por semana    2. Dos a tres veces por mes    3. Una vez por mes    4. Dos o tres veces al año    5. Casi nunca    6. Nunca | | |

| SECCIÓN 2: LAS ACTITUDES SOBRE LA ENTREGA DE MÉTODOS ANTICONCEPTIVOS | | | | | | | | |
| --- | --- | --- | --- | --- | --- | --- | --- | --- |
| 1. **¿Cuál es su opinión con respecto de si los/as médicos y matrones/as deberían proveer información y/o servicios anticonceptivos a los siguientes grupos de adolescentes…?** | | | | | | | | |
|  | Nunca es apropiado con o sin el consentimiento de los padres | | Nunca es apropiado sin el consentimiento de los padres | | A veces es apropiado sin el consentimiento de los padres | | Siempre es apropiado sin el consentimiento de los padres | |
| - 1. **Informar** sobre anticonceptivos a adolescentes entre **14-17 años** |  | |  | |  | |  | |
| - 1. **Proveer** de anticonceptivos a adolescentes entre **14-17 años** |  | |  | |  | |  | |
| - 1. Informar sobre anticonceptivos a adolescentes **menores de 14 años** |  | |  | |  | |  | |
| - 1. **Proveer** de anticonceptivos a adolescentes **menores de 14 años** |  | |  | |  | |  | |
| SECCIÓN 3: INTENCIONES A PROVEER ABORTO | | | | | | | | |
| 1. **Ahora que el aborto es legal en algunas circunstancias ¿cómo cree que va a afectar su futuro ejercicio profesional…?** | | | | | | | | |
|  | | **Muy en desacuerdo** | | **En desacuerdo** | | **De acuerdo** | | **Muy de acuerdo** |
| - 1. Me voy a capacitar para prestar servicios de aborto | |  | |  | |  | |  |
| - 1. Derivaré a mujeres que desean abortar a otros médicos que las puedan atender | |  | |  | |  | |  |
| - 1. Trataré de disuadir a las mujeres que deseen abortar | |  | |  | |  | |  |
| - 1. Trataré de convencer a otros médicos de realizar abortos | |  | |  | |  | |  |
| - 1. No voy a realizar un aborto bajo ninguna circunstancia | |  | |  | |  | |  |

| SECCIÓN 4: RAZONES PERSONALES PARA NO PROVEER SERVICIOS DE ABORTO | | | | | | | |  |
| --- | --- | --- | --- | --- | --- | --- | --- | --- |
| 1. **Ahora que el aborto es legal en algunas circunstancias ¿Hasta qué punto cree que los siguientes factores le preocuparían acerca de practicar un aborto?** | | | | | | | |  |
|  | **Muy en desacuerdo** | **En desacuerdo** | | **Ni acuerdo ni en desacuerdo** | | **De acuerdo** | **Muy de acuerdo** |  |
| - 1. Está en contra de mis creencias religiosas |  |  | |  | |  |  |  |
| - 1. Estaría fuera de mi deber profesional |  |  | |  | |  |  |  |
| - 1. Está en contra de mis valores personales |  |  | |  | |  |  |  |
| - 1. Tendría miedo de ser rechazado/a por mis colegas y/o estigmatizado/a en mi profesión |  |  | |  | |  |  |  |
| - 1. Tendría miedo de ser rechazado/a por mi familia o grupos de amigos |  |  | |  | |  |  |  |
| - 1. Tendría miedo de que hostiguen a mi familia o a mí |  |  | |  | |  |  |  |
| - 1. Tendría miedo de tener problemas legales |  |  | |  | |  |  |  |
| SECCIÓN 5: LAS ACTITUDES SOBRE EL ABORTO | | | | | | | | |
| 1. **Para cada una de las siguientes situaciones, ¿me podría decir si está de acuerdo o en desacuerdo con que la ley despenalice el aborto, o sea, que no castigue con pena de cárcel a la mujer si se realiza un aborto…?** | | | | | | | | |
|  | | | **En desacuerdo** | | **De acuerdo** | | | |
| - 1. Si una mujer no desea tener un hijo | | |  | |  | | | |
| - 1. Si una pareja en conjunto decide no tener un hijo | | |  | |  | | | |
| - 1. Si la vida de la madre corre serio peligro por el embarazo | | |  | |  | | | |
| - 1. Si el feto tiene un serio defecto que lo hace inviable, o sea, que es seguro que morirá antes de nacer o al poco tiempo de nacer | | |  | |  | | | |
| - 1. Si la mujer quedó embarazada producto de una violación | | |  | |  | | | |
| - 1. Si la mujer o pareja no tiene los medios económicos para criar al hijo | | |  | |  | | | |
| - 1. Si la madre es menor de 14 años | | |  | |  | | | |

| 1. **¿Cuán de acuerdo o en desacuerdo está con las siguientes afirmaciones sobre el aborto…?** | | | | | | | | | | | | | | | |
| --- | --- | --- | --- | --- | --- | --- | --- | --- | --- | --- | --- | --- | --- | --- | --- |
|  | | **Muy en desacuerdo** | | | **En desacuerdo** | | | **De acuerdo** | | **Muy de acuerdo** | | | | **No sé** | |
| - 1. El aborto es aceptable en cualquier situación | |  | | |  | | |  | |  | | | |  | |
| - 1. El aborto es aceptable si la mujer no tiene el apoyo de la familia | |  | | |  | | |  | |  | | | |  | |
| - 1. El aborto es aceptable si la mujer tiene una discapacidad intelectual | |  | | |  | | |  | |  | | | |  | |
| - 1. El aborto es aceptable si la mujer tiene una discapacidad sensorial (por ejemplo es ciega o sorda) | |  | | |  | | |  | |  | | | |  | |
| - 1. El aborto es aceptable si el hombre no apoya a la mujer en tener el hijo | |  | | |  | | |  | |  | | | |  | |
| - 1. El aborto es aceptable si la mujer considera que ya tiene suficientes hijos | |  | | |  | | |  | |  | | | |  | |
| - 1. El aborto es un derecho de la mujer | |  | | |  | | |  | |  | | | |  | |
| - 1. El aborto siempre es malo | |  | | |  | | |  | |  | | | |  | |
| - 1. El aborto puede ser algo bueno para algunas mujeres en algunas situaciones | |  | | |  | | |  | |  | | | |  | |
| - 1. El aborto es la manera fácil de salir de un embarazo no planificado | |  | | |  | | |  | |  | | | |  | |
| - 1. El aborto es igual a un homicidio | |  | | |  | | |  | |  | | | |  | |
| - 1. El aborto es aceptable pasado el primer trimestre de embarazo (13+ semanas) en situaciones excepcionales | |  | | |  | | |  | |  | | | |  | |
| - 1. El aborto es aceptable pasado el primer trimestre de embarazo (13+ semanas) en cualquier situación | |  | | |  | | |  | |  | | | |  | |
| 1. **¿Cuán de acuerdo o en desacuerdo están sus compañeros de carrera con las siguientes afirmaciones sobre el aborto…?** | | | | | | | | | | | | | | | |
|  | | | **Muy en desacuerdo** | | | **En desacuerdo** | | | **De acuerdo** | | | **Muy de acuerdo** | | | **No sé** |
| - 1. El aborto es un derecho de la mujer | | |  | | |  | | |  | | |  | | |  |
| - 1. El aborto siempre es malo | | |  | | |  | | |  | | |  | | |  |
| - 1. El aborto es la manera fácil de salir de un embarazo no planificado | | |  | | |  | | |  | | |  | | |  |
| - 1. El aborto puede ser algo bueno para algunas mujeres en algunas situaciones | | |  | | |  | | |  | | |  | | |  |
| - 1. El aborto es igual al homicidio | | |  | | |  | | |  | | |  | | |  |
| 1. **Por favor señale el grado en que usted personalmente está de acuerdo o en desacuerdo con las siguientes afirmaciones:** | | | | | | | | | | | | | | | |
|  | **Muy en desacuerdo** | | | **En desacuerdo** | | | **Ni acuerdo ni en desacuerdo** | | | | **De acuerdo** | | **Muy de acuerdo** | | |
| - 1. Las necesidades de los pacientes son más importantes que las creencias de los/las médicos/obstetras |  | | |  | | |  | | | |  | |  | | |
| - 1. Los/as médicos/obstetras tienen la responsabilidad de aconsejar a sus pacientes en contra del aborto |  | | |  | | |  | | | |  | |  | | |
| - 1. Brindar servicios de aborto es una contribución positiva para la sociedad |  | | |  | | |  | | | |  | |  | | |
| - 1. Brindar servicios de aborto es erróneo moralmente |  | | |  | | |  | | | |  | |  | | |
| - 1. El aborto debe ser gratuito como parte de las prestaciones de la salud pública |  | | |  | | |  | | | |  | |  | | |

| SECCIÓN 6: ACTITUDES SOBRE LA OBJECIÓN DE CONCIENCIA | | | | | | | | | | |
| --- | --- | --- | --- | --- | --- | --- | --- | --- | --- | --- |
| 1. **Las siguientes afirmaciones se refieren a diferentes aspectos de la objeción de conciencia. Para cada uno, indique si usted está en desacuerdo o de acuerdo.** | | | | | | | | | | |
|  | **Muy en desacuerdo** | | **En desacuerdo** | | **Ni acuerdo ni en desacuerdo** | | **De acuerdo** | | **Muy de acuerdo** | |
| - 1. Los/as médicos objetores de conciencia debieran informarle a las pacientes con posibilidades de aborto legal de todas sus opciones, incluido el aborto |  | |  | |  | |  | |  | |
| - 1. Los/as médicos objetores de conciencia debieran derivar a las pacientes con posibilidades de aborto legal a un/a médico que esté dispuesto a realizarlo |  | |  | |  | |  | |  | |
| - 1. Los/as médicos debieran poder recurrir a la objeción de conciencia para evitar dar atención postaborto |  | |  | |  | |  | |  | |
| - 1. Yo usaría la objeción de conciencia para evitar atender a una mujer con complicaciones postaborto |  | |  | |  | |  | |  | |
| - 1. Yo usaría la objeción de conciencia para evitar atender a una mujer que quisiera abortar, sin importar sus razones |  | |  | |  | |  | |  | |
| - 1. Yo usaría la objeción de conciencia para evitar atender a una mujer que quisiera un aborto legal |  | |  | |  | |  | |  | |
| - 1. A nivel personal generalmente apoyo la *objeción de conciencia* |  | |  | |  | |  | |  | |
| 1. **Específicamente: ¿Qué profesionales/ técnicos/ trabajadores de la salud deben tener derecho a ejercer objeción de consciencia?**   [Seleccione todas las respuestas que correspondan]   - 1. Enfermeros/as   2. Matrones/as   3. Médicos/as   4. Farmacéuticos/as   5. Personal administrativo   6. Otro: _______   7. Ninguno | | | | | | | | | | |
| 1. **En su opinión personal, ¿cuán de acuerdo está con implementar las siguientes formas de regulación de la objeción de conciencia en Chile?** | | | | | | | | | | |
|  | | **Muy en desacuerdo** | | **En desacuerdo** | | **Ni acuerdo ni en desacuerdo** | | **De acuerdo** | | **Muy de acuerdo** |
| - 1. Registro **público** obligatorio de objetores de conciencia | |  | |  | |  | |  | |  |
| - 1. Las universidades y otras **instituciones** debieran poder registrarse como objetores de conciencia | |  | |  | |  | |  | |  |

| SECCIÓN 7: ACTITUDES SOBRE LA DENUNCIA Y LA CONFIDENCIALIDAD | | | | | |
| --- | --- | --- | --- | --- | --- |
| 1. **Considerando el marco regulatorio actual ¿cuál es su opinión sobre las siguientes situaciones…?** | | | | | |
| **Un/a médico y/o matrón/a trabajando en……** | **Muy en desacuerdo** | **En desacuerdo** | **De acuerdo** | | **Muy de acuerdo** |
| - 1. …un **centro de salud público** debería denunciar a una niña menor de 14 años que está teniendo relaciones sexuales consentidas con otro menor de edad |  |  |  | |  |
| - 1. ….una **clínica privada** debería denunciar a una niña menor de 14 años que está teniendo relaciones sexuales consentidas con otro menor de edad |  |  |  | |  |
| - 1. …un **centro de salud público** debería denunciar a una mujer si **sospecha** que ella se realizó un aborto **ilegalmente** |  |  |  | |  |
| - 1. ….una **clínica privada** debería denunciar a una mujer si **sospecha** que ella se realizó un aborto **ilegalmente** |  |  |  | |  |
| - 1. ….**un centro de salud público** debería denunciar a una mujer si **confirma** que ella se realizó un aborto **ilegalmente** |  |  |  | |  |
| - 1. ….una **clínica privada** debería denunciar a una mujer si **confirma** que ella se realizó un aborto **ilegalmente** |  |  |  | |  |
| Un/a **médico** y/o **matrón/a** que… | **Muy en desacuerdo** | **En desacuerdo** | **De acuerdo** | **Muy de acuerdo** | |
| - 1. …realiza un aborto **quirúrgico y seguro**, pero **ilegal** debe ir preso/a |  |  |  |  | |
| - 1. …entrega o receta **pastillas** (misoprostol) para que una mujer se pueda **realizar un aborto ilegalmente** debe ir preso/a |  |  |  |  | |
| - 1. ….atiende una mujer que se realizó un aborto **ilegalmente** tiene la obligación de mantener esta información en forma **confidencial** |  |  |  |  | |
| **Una mujer…** | **Muy en desacuerdo** | **En desacuerdo** | **De acuerdo** | **Muy de acuerdo** | |
| - 1. …que se realizó un aborto **ilegalmente** debe **ir presa** |  |  |  |  | |
| - 1. …debe tener acceso a **información** sobre cómo hacerse un aborto seguro con pastillas |  |  |  |  | |

| SECCIÓN 8: EXPERIENCIA Y CONOCIMIENTO PERSONAL | | | | | |  |
| --- | --- | --- | --- | --- | --- | --- |
| 1. **¿Cuántas veces usted ha estado embarazada o ha embarazado a alguien? ________**    1. [si respuesta >0]: ¿Cuántos de esos embarazos terminaron en...?       1. El nacimiento de una guagua: ___       2. Un aborto espontáneo/nacimiento de un bebé muerto: ___       3. Un aborto inducido: __ | | | | | |  |
| SECCIÓN 9: MEJOR AMIGA | | | | | |  |
| Ahora, me gustaría que pensara en su mejor amiga en Chile. Imagínela en su mente. | | | | | |  |
| 1. **Su mejor amiga, ¿ha tenido un aborto inducido alguna vez?**    1. Sí    2. Sospecho que ha tenido un aborto    3. No    4. No sé | | | | | |  |
| 1. [27=A o B]: **¿Dónde lo hizo?**    1. Chile    2. Uruguay    3. Argentina    4. Perú    5. México    6. EE.UU.    7. Otro país: _______    8. No sé | | | | | |  |
| 1. [27=A o B]: **¿Hace cuánto tiempo cree que su mejor amiga lo hizo?**    1. Durante el último año    2. Durante los últimos 5 años    3. Hace más de 5 años    4. No sé | | | | | |  |
| 1. [27=A o B]: **¿Qué procedimiento siguió? Marque todas las casillas que aplican:**    1. Consultó a un médico u obstetra    2. El procedimiento fue realizado por un médico u otro profesional de la salud    3. El procedimiento fue realizado por una persona que no es profesional de la salud    4. Fue a un centro de salud público    5. Fue a una clínica privada    6. Fue a una lugar clandestino    7. Usó Misoprostol    8. Usó anticonceptivos de emergencia antes de saber si estaba embarazada    9. Usó anticonceptivos de emergencia después de confirmar que estaba embarazada    10. Usó algún otro medicamento o droga    11. Usó hierbas    12. Se pegó en el abdomen    13. Otra cosa: ______________    14. No sé | | | | | |  |
| 1. [27=A o B]: **¿Resultó en un aborto completo?**    1. Sí    2. No    3. No sé | | | | | |  |
| SECCIÓN 10: LA EDUCACIÓN SEXUAL ESCOLAR | | | | | | |
| 1. **Pensando en su educación escolar, ¿cuán de acuerdo se encuentra con la siguiente afirmación?**   “*Durante mi educación escolar me enseñaron en enseñaron en clases, charlas o talleres cómo evitar el contagio de enfermedades de transmisión sexual*”   - 1. Muy en desacuerdo   2. En desacuerdo   3. De acuerdo   4. Muy de acuerdo | | | | | | |
| 1. **Pensando en tu educación escolar, ¿Existía algún programa de educación sexual en su establecimiento?** 2. Sí 3. No 4. No sé | | | | | | |
| 1. Pensando en una escala del 1 al 7, donde 1 es pésima y 7 es excelente, **¿Cómo calificaría la educación sexual que recibió en su establecimiento escolar?**   (Pésima) 1 2 3 4 5 6 7 (Excelente) | | | | | | |
| SECCIÓN 11: ENSEÑANZA DEL ABORTO | | | | | | |
| 1. **¿Cuál de las siguientes palabras/conceptos cree que representa de mejor manera el sello de su universidad en temas relacionados a la salud sexual y reproductiva?** | | | | | | |
|  | **Muy en desacuerdo** | **En desacuerdo** | **Ni acuerdo ni en desacuerdo** | **De acuerdo** | **Muy de acuerdo** | |
| 1. Pluralista (diversidad de perspectivas) |  |  |  |  |  | |
| 1. Enfocada en los derechos de la mujer |  |  |  |  |  | |
| 1. Enfocada en la defensa de la vida del feto |  |  |  |  |  | |
| 1. Laica |  |  |  |  |  | |
| 1. Doctrina católica |  |  |  |  |  | |
| 1. Homogénea (hay una sola perspectiva) |  |  |  |  |  | |
| 1. **¿Qué tan de acuerdo está con la forma en que se enseña la salud sexual y reproductiva en su Universidad?** 2. Muy de acuerdo 3. De acuerdo 4. En desacuerdo 5. Muy en desacuerdo | | | | | | |
| 1. **Pensando en una escala del 1 al 7, donde 1 es pésima y 7 es excelente, ¿Cómo evaluaría la enseñanza en temas de salud sexual y reproductiva que recibe en su Universidad?**   (Pésima) 1 2 3 4 5 6 7 (Excelente) | | | | | | |
| 1. **Ahora que el aborto es legal en algunas circunstancias, ¿a quienes cree que su universidad debería capacitar en prestar servicios de aborto?**   [Seleccione todas las respuestas que correspondan]   1. Estudiantes de medicina en general 2. Estudiantes de medicina especializándose en ginecología 3. Estudiantes de obstetricia 4. Otro: _____________ 5. Ningún estudiante | | | | | | |
| 1. **¿Cree que debería cambiar en algo el contenido de los cursos de salud sexual y reproductiva de su carrera con la despenalización del aborto?** 2. Sí 3. No | | | | | | |
| SECCIÓN 12: PREGUNTAS FINALES ABIERTAS | | | | | | |
| 1. **¿Qué le recomendaría a su Universidad para mejorar la enseñanza que entrega sobre temas de salud sexual y reproductiva?** | | | | | | |
| 1. **¿Qué sentimientos u opiniones tiene acerca del estatus legal del aborto en Chile?** | | | | | | |
| 1. **¿Cómo recibió esta encuesta?**   [Seleccione todas las respuestas que correspondan]   - 1. Fue enviada por mi profesor   2. Fue enviada por el centro de alumnos   3. Un/a compañero/a de clase me la mostró   4. Facebook   5. Otro sitio web: ____________   6. Otro: _____________ | | | | | | |
| 1. **¿Cómo se sintió completando esta encuesta?**    1. Me sentí bien    2. No me sentí ni bien ni mal    3. Me sentí mal | | | | | | |

| **Le agradecemos sus respuestas y solo tenemos unas preguntas más para usted. Estas preguntas son para poder participar de un sorteo de regalos y para señalarnos si desea recibir copia de la eventual publicación de este estudio. Recuerde que este estudio es anónimo – si no desea compartir su información de contacto con nosotros, simplemente ignore el siguiente formulario. La participación es completamente voluntaria.**  **El siguiente es un formulario separado. Cualquier información de contacto que nos proporcione no será vinculada con sus respuestas a la encuesta.** |
| --- |
| Para decir gracias, tenemos un regalo para nuestros participantes:  ¡El sorteo de un premio!  ¡Usted podría ganar una de las **25** tarjetas de regalo por **20.000 pesos**!  **¿Desea participar en el sorteo?** Sólo se le contactará con respecto a la rifa si gana.   - Sí - No |
| [si Sí]:  Indique su dirección de correo electrónico: _______________________________ |
| Cuando este estudio termine, **¿desea recibir un resumen ejecutivo de sus resultados y/o una copia de la eventual publicación que se realice?**   - Sí - No |
| [si Sí]:  Indique su dirección de correo electrónico: _______________________________ |
